# Visfatin, glucose metabolism and vascular disease: a review of evidence

**DOI:** 10.1186/1758-5996-2-21

**Published:** 2010-03-26

**Authors:** Pedro Saddi-Rosa, Carolina SV Oliveira, Fernando MA Giuffrida, André F Reis

**Affiliations:** 1 Federal University of São Paulo, Escola Paulista de Medicina, Diabetes Center, São Paulo, Brazil; 2 Federal University of Bahia, Internal Medicine Department, Salvador, Brazil

## Abstract

The adipose tissue is an endocrine organ producing substances called adipocytokines that have different effects on lipid metabolism, metabolic syndrome, and cardiovascular risk. Visfatin was recently described as an adipocytokine with potentially important effects on glucose metabolism and atherosclerosis. Visfatin has been linked to several inflammatory conditions, beta cell function, and cardiovascular disease. The growing number of publications on the subject shall bring further evidence about this adipocytokine. Its findings may contribute in the identification of higher risk individuals for diabetes and cardiovascular disease with a better comprehension about the complex intercorrelation between adiposity, glucose metabolism and vascular disease.

## Introduction

The ongoing global epidemic of atherosclerotic disease is propelled by a concurrent rise in the prevalence of obesity, insulin resistance, and type 2 diabetes (T2DM). In this setting, the coexistence of several cardiovascular risk factors aggregating around the metabolic syndrome (MS), such as hyperglycemia, hypertension, dyslipidemia and visceral obesity, is well-known [1]. The pathophysiological relationship between cardiovascular disease and these risk factors is of great importance due to their high morbidity and mortality as well as impact on healthcare costs [2]. Therefore, the link between visceral adiposity and insulin resistance, endothelial dysfunction, and subsequent atherosclerosis has been extensively studied.

Adipose tissue is a known endocrine organ secreting several soluble factors, known as adipocytokines or adipokines, some of these being adiponectin, leptin, resistin and, more recently, visfatin. They can partly explain the link between obesity, insulin resistance, beta-cell dysfunction, endothelial dysfunction, and atherosclerosis [3]. Other known products of adipocytes are free fatty-acids, tumor necrosis factor (TNF)-alpha, interleukin (IL)-6, IL-1, Monocyte chemoattractant protein (MCP)1, coagulation mediators such as platelet-activator inhibitor (PAI)-1, and complement components [1].

Atherosclerosis is a complex inflammatory disease, involving many physiological mechanisms, in which immunologic, metabolic, genetic, and environmental factors interact. The endothelium plays a pivotal role in these interactions, through disequilibrium in regulation of vascular tonus (endothelium-dependent relaxation), platelet aggregation, coagulation, and fibrinolysis [4]. Risk factors for this condition have been continuously identified.

Adipocytokines may have a role in each one of these processes, explaining at least in part the association between obesity, insulin resistance, diabetes, and cardiovascular disease.

## Visfatin

Visfatin is an adipokine identified in 2004 [5] and thus named for the suggestion that it would be predominantly produced and secreted in visceral fat. Visfatin is highly preserved across animal evolution. It has a molecular weight of 52 KDa and its gene encodes 491 aminoacids. It is identical to pre-B cell colony-enhancing factor (PBEF), described in 1994 as a cytokine produced by lymphocytes, acting on lymphocyte maturation and inflammatory regulation. Visfatin was also soon recognized as the formerly described Nicotinamide phosphoribosyltransferase (Nampt), the limiting enzyme in nicotinamide adenine dinucleotide (NAD) biosynthesis. In addition to being produced in human leukocytes and adipose tissue, visfatin is also expressed in human and animal hepatocytes and muscles [6,7] and in animal adipocytes, kidney and heart [8-10]. Visfatin was found to be released predominantly from macrophages rather than from adipocytes in visceral adipose tissue. In this regard, there is sufficient evidence to consider that visfatin is expressed by the macrophages infiltrating adipose tissue and is produced in response to inflammatory signals [11,12]. It is now believed that visfatin actions can be endocrine, paracrine, and autocrine as well. These autocrine effects of visfatin may play an important role in regulating insulin sensitivity in the liver [9].

Fukuhara et al. investigated genes having diverse patterns of expression in subcutaneous and visceral fat from two human subjects, identifying one gene with this feature [5]. Sequencing revealed this gene to correspond to PBEF. Increased visfatin expression and secretion was found during cell differentiation of cultured adipocytes. Moreover, studying 101 humans, strong correlation between visceral fat estimated by computed tomography (CT) and visfatin plasma levels was found, as well as a weak correlation between visfatin levels and subcutaneous fat. Furthermore, an increased visceral fat expression of visfatin was demonstrated after weight gain in obese diabetic mice. In these animals, acute administration of recombinant visfatin decreased plasma glucose in a dose-dependent fashion, in both insulin-resistant and insulin-deficient animals. Chronic increase of visfatin expression obtained by viral transfection has also decreased glucose in both animal models. A homozygous knockout model for the visfatin gene has been attempted, but it was incompatible with life, fetal death occurring in the initial phase of embryogenesis. However, heterozygous animals showed plasma levels of visfatin two-thirds of those seen in wild-type animals, associated to blood glucose slightly higher in fasting, post-prandial state, and after an oral glucose load. In addition, the study has demonstrated insulin-mimetic effects of visfatin such as: increased glucose uptake in adipocytes and myocytes, suppression of hepatic glucose release, stimulated triglyceride accumulation, and increase in its synthesis in pre-adipocytes.

Further on, the authors investigated the intracellular signaling of visfatin and suggested it to tyrosine-phosphorylate the insulin receptor in hepatocytes, myocytes, and adipocytes. Competitive inhibition testing showed that both molecules do not compete for the insulin receptor, raising the hypothesis that visfatin could have a binding site different from that of insulin. Actually, recombinant receptors with structural changes decreasing affinity for insulin didn't change visfatin binding.

This study has opened a broad perspective for the role of visfatin in mechanisms of glucose control. Although its insulin-mimetic effects have been questioned, generating a retraction by the author in 2007 [13], numerous subsequent studies demonstrated visfatin to bear important metabolic effects as discussed below.

## General actions of Visfatin and correlations with medical conditions

Subsequent publications have reported various effects and correlations between visfatin plasma levels with various medical conditions (Table 1). It possesses anti-apoptotic effects on neutrophils in both animal and clinical models of sepsis [14]. It is also increased in acute pulmonary lesion, being useful as a marker of this condition [15]. Visfatin is also diminished in patients with steatohepatitis when compared to pure steatosis [16]. However, increased visfatin levels correlated positively with portal inflammation [17]. These observations could suggest an association of visfatin with inflammation. Negative correlation of visfatin with creatinine clearance and positive correlation with urinary albumin excretion has been demonstrated, suggesting visfatin circulating levels to be influenced by renal function [18]. An inhibitor of visfatin (FK866) is being tested as a potential anti-tumor agent due to NAD depletion leading to apoptosis on highly metabolically active cells [19].

**Table 1:** Association between several medical conditions and visfatin

Medical Condition	Visfatin
Atherosclerosis	Increased serum levels [10,31] Increased expression in instable plaques [31]

Endothelial dysfunction	Increased serum levels [18]

Cardioprotection	Reduces infarct area [36]

Metabolic Syndrome	Controversial, Increased serum levels [34]

Adiponectin	Controversial [18,27]

Hepatic Fat Disease	Diminished serum levels in steatohepatitis [16] Increased serum levels with progressive portal inflammation [17]

Tumoral replication	Visfatin antagonist being tested [19]

Acute lung injury	Increased serum levels [15]

Renal insufficiency	Increased serum levels [18]

Beta cell function impairment	Increased serum levels [10,22,23,28,29]

Insulin resistance	No correlation [20,22,23]

Insulin-mimetic effect	Controversial, no effect [5,10,15-20,22-24,27-29]

Diabetes	Controversial [18,23,27,28]

Obesity	Increased serum levels [20,22]

Visceral Fat	Controversial, no correlation [5,15-20,27]

Vascular smooth muscle	Promotes inflammation [32]

The relationship of visfatin with visceral fat has been questioned. Relationship of plasma visfatin levels was seen with body mass index (BMI) and percentage of body fat but not with abdominal circumference or visceral fat estimated by CT [20]. Chang et al. did not find different expression of visfatin between visceral and subcutaneous adipose tissue in 53 Taiwanese adults [21]. Nevertheless, there is strong evidence that visfatin increases with obesity as demonstrated in a prospective cohort study, in which visfatin levels were augmented in morbidly obese subjects compared to normal-weight individuals. Visfatin normalized after 6 months of bariatric surgery and consequent weight loss [22]. Altogether current evidence does not support the notion that visfatin is predominantly expressed in visceral fat [21].

## Visfatin in glucose metabolism

After the study by Fukuhara suggested a role of visfatin on glucose metabolism, others have investigated its relationship with T2DM, insulin secretion and sensitivity, and also with other adipokines such as adiponectin.

Revollo et al. showed that visfatin is an essential enzyme in NAD production (Nampt). It exists both in intra and extracellular environments. Mice heterozygous for mutations in the visfatin gene have glucose intolerance mainly due to insulin secretion deficiency. This insulin secretion defect can be corrected by administering nicotinamide mononucleotide (NMN), the product of visfatin on NAD biosynthesis. Since the pancreas has very low levels of intracellular visfatin, the author suggests that maintenance of high NMN circulating levels by extracellular visfatin would be critical for normal beta-cell function [10]. Skop et al. investigated glucose uptake in animal hepatocytes with diminished visfatin expression. They found significantly reduced NAD biosynthesis and significantly decreased incremental glucose uptake after stimulation with insulin when compared to control hepatocytes with normal expression of visfatin [9]. A negative correlation of visfatin levels with beta-cell function was demonstrated by studying acute insulin secretion assessed by an intravenous glucose tolerance test [23]. Furthermore, continuous glucose infusion in humans acutely increases visfatin levels. This effect is suppressed by insulin or somatostatin infusion [24].

Brown and co-authors demonstrated that incubation with visfatin into mouse pancreatic beta-cells caused significant changes in the mRNA expression of several key diabetes-related genes, including marked up-regulation of hepatocyte nuclear factor 1 homeobox B (HNF1B) among others. Visfatin also caused a significant 46% increase in insulin secretion compared to control at low glucose, and this increase was blocked by co-incubation with the specific visfatin inhibitor FK866. Both visfatin and NMN induced activation of insulin receptor and extracellular signal-regulated kinase (ERK)1/2, with visfatin-induced activation of insulin receptor and ERK1/2 being inhibited by FK866. The authors suggested that visfatin can significantly regulate insulin secretion, insulin receptor phosphorylation, and expression of a number of genes associated with beta-cell function in mice [25].

Visfatin levels were also investigated in some population based studies. Chen et al., investigating visfatin levels in a Chinese population, found a positive association between visfatin and the presence of T2DM, even after adjustment for BMI, age, sex, smoking status, blood pressure, and lipid profile [26]. Dogru et al., studying visfatin levels in 40 subjects with newly diagnosed diabetes or glucose intolerance, found that visfatin levels were higher in the diabetic patients when compared to controls, but not when compared to glucose intolerant patients (pre-diabetes)[27]. Patients with both long duration type 1 diabetes (T1DM) and T2DM had higher visfatin levels compared to non-diabetic controls or recently diagnosed diabetic individuals. In T2DM, visfatin levels were partly associated to higher glycated hemoglobin levels (A1C) [23]. Nevertheless, Takebayashi found no correlation between diabetes and visfatin [18], while another study, demonstrated decreased visfatin in patients with T1DM and inverse relationship between A1C and visfatin levels [28].

The relationship between visfatin levels and insulin resistance surrogates was also investigated. However, it was not possible to demonstrate correlation of visfatin with HOMA-IR [20,23]. HOMA-IR also did not show correlation with visfatin expression on visceral adipose tissue but was positively associated with visfatin expression in subcutaneous adipocytes [21]. Furthermore, administration of the insulin sensitizer pioglitazone for 3 weeks didn't affect plasma levels of visfatin in humans with recently diagnosed T2DM [29]. The same drug administered for 12 weeks did not change visfatin levels either [18]. This is not, apparently, a general effect of thiazolidinediones, since rosiglitazone administered for 3 weeks increased visfatin levels in normoglycemic individuals [30].

Current data suggests that visfatin is important to normal insulin secretion, but its relationship with diabetes risk and progression is still a matter of debate. Thus, visfatin may be a compensatory mechanism or part of the pathophysiology of diabetes.

## Cardiovascular disease and visfatin

Oxidized LDL was verified to increase visfatin expression in cultured monocytes. Moreover, visfatin increased the expression of molecules that degrade extracellular matrix causing plaque instability. This later effect was abolished after adding an inhibitor of insulin receptor signaling [31].

Visfatin expression is increased in unstable coronary plaques and symptomatic carotid lesions in humans. Patients undergoing carotid endarterectomy and presenting ischemic symptoms 6 months prior to evaluation had higher visfatin expression than asymptomatic individuals. The same finding has been replicated in patients with unstable angina compared to those with stable angina. Immunohistochemical analysis of the plaques revealed higher concentration of visfatin in their lipid nucleuses compared to stable plaques. The same study showed that balloon angioplasty, acutely injuring the plaques, caused visfatin levels to peak 4 hours after the procedure, receding back to basal levels after 24 hours. Another study found a negative correlation between visfatin and endothelial function evaluated through flow-mediated or nitroglycerin-mediated dilation [18]. Visfatin can promote vascular smooth muscle inflammation, being associated with a potential role in vascular dysfunction and inflammation associated with some metabolic disorders [32].

Increased visfatin levels are associated to coronary artery disease (CAD) and acute coronary syndromes even after correction for classic cardiovascular risk factors such as cholesterol, smoking, hypertension, diabetes, and obesity [33]. A positive association between visfatin and MS was noted, mainly among individuals with carotid atherosclerosis. Indeed, visfatin was an independent risk factor for augmented carotid intimal-media thickness [34]. In this regard, it is well established that acute ischemic stroke is another possible consequence of atherosclerosis. Recently, higher visfatin levels were found in Chinese individuals with stroke, suggesting a possible role in this vascular disease [35].

A cardioprotective effect of visfatin was also suggested in an animal model of non-atherosclerotic ischemia and reperfusion [36]. Visfatin administered at the moment of reperfusion diminished the size of the infarcted area. This effect was mediated by the inhibition of the mitochondrial permeability transition pore (mPTP) opening, a non-specific mitochondrial channel, its opening in the first few minutes of myocardial reperfusion being a critical determinant of cardiomyocyte death. The authors emphasize that this was an acute effect and chronically elevated levels of visfatin may yield different results.

## Genetic studies

The visfatin/PBEF gene consists of 11 exons and 10 introns spanning 34.7-kb and is located on chromosome 7q22.2. Few studies of polymorphic markers in the visfatin gene have been reported to date. Bottcher et al. [37] did not find any association with either T2DM, in a cohort of 503 diabetic subjects and 476 healthy controls, or with T2DM-related traits in 626 non-diabetic subjects from Germany. However, they found an association between the -948 G > T single-nucleotide polymorphism (SNP) and fasting insulin levels in non-diabetic subjects (*p *< 0.05). The ratio of visceral/subcutaneous visfatin mRNA expression was associated with all three genetic polymorphisms studied (rs9770242, -948G > T, rs4730153).

To investigate the role of visfatin gene variants in obesity-related phenotypes, Bailey et al. [38] genotyped a total of 13 SNPs in the promoter region of the gene in 918 participants from 208 families evaluated in the Quebec Family Study. A significant association was found between two SNPs (rs9770242 and rs1319501) and fasting insulin levels (*p *= 0.002). These SNPs were also associated with fasting glucose (*p *= 0.02). Furthermore, they found that a more distal SNP (rs7789066) was significantly associated with the apolipoprotein B component of VLDL (*p *= 0.012).

Zhang et al. [39] studied a group of 814 white patients from the USA and a group of non-diabetic controls (n = 320). They found a significant association between -948C > A and T2DM (*p *= 0.021). In a non-diabetic population (n = 630), the same -948C allele that conferred increased risk of T2DM was significantly associated with higher plasma levels of fibrinogen and C-reactive protein (*p *= 0.0022 and 0.0038, respectively). However, no significant associations were observed with BMI, waist circumference, serum glucose levels, or fasting insulin levels. They suggested that the visfatin gene may play a role in determining T2DM susceptibility, possibly by modulating chronic, low-grade inflammatory responses.

Korner et al. [40] studied 731 schoolchildren and an independent cohort of 167 obese children from Germany. They genotyped 3 SNPs (rs9770242, -948G > T, rs4730153). The authors did not find association of any of the 3 polymorphisms or their haplotypes with BMI, waist-to-hip ratio, glucose, insulin, or lipid levels. However, the -948G variant was associated with significantly higher diastolic blood pressure in obese children (*p *< 0.05 after adjusting for age, sex, pubertal stage, and height).

Blakemore et al. [41] studied 1,709 severely obese subjects together with 2,367 T2DM individuals and 2,850 controls. For quantitative trait analysis, an additional 2,362 subjects were typed for rs10487818 from a general population sample. This rare SNP, rs10487818, located in intron 4, was associated with protective effect against severe obesity. This is one of the first rare SNPs shown to be protective against a common polygenic disease like obesity and provides further evidence that rare alleles of strong effect can contribute to this kind of complex diseases.

More recently, Yan et al. [42] suggested that Visfatin -1535C > T polymorphism might be associated with reduced risk of CAD in a Chinese population. There was a significant association between Visfatin -1535C > T polymorphism and triglyceride levels in both CAD patients and controls (*p *= 0.003). In non diabetic Japanese subjects, Tokunaga et al. [43] found that the -1535T/T genotype modulates lipid levels promoting lower serum triglyceride levels and higher HDL-cholesterol levels. In this investigation, reporter gene assay of 3T3-L1 adipocytes revealed that the promoter activity of -1535T and -1535C was similar, suggesting that the observed association may reflect linkage disequilibrium between -1535T > C and other causative variations of the visfatin gene.

Taken together, these studies suggest that genetic variants in the Visfatin gene can be associated with some phenotypes related to glucose, lipids, and other metabolic and vascular traits. Further studies in other populations must be carried out to better clarify these points.

## Conclusion and perspectives

Visfatin is is an adipokine involved in inflammatory phenomena, atherosclerosis, and possibly in insulin secretion. Its physiology has begun to be described but its role on diabetes and CAD pathophysiology is still controversial.

New studies may define the possible role of visfatin in the mechanisms of glycemic control, vascular function and atherosclerotic process. These findings may contribute in the identification of higher risk individuals as well as lead to new therapeutic approaches. Genetic studies about the visfatin gene and its variations will also contribute to the elucidation of these phenomena.

## List of abbreviations

BMI: body mass index; CAD: coronary artery disease; CT: computed tomography; mPTP: mitochondrial permeability transition pore; MS: metabolic syndrome; NAD: nicotinamide adenine dinucleotide; Nampt: nicotinamide phosphoribosyltransferase; NMN: nicotinamide mononucleotide; PBEF: pre-B cell colony-enhancing factor; SNP: single-nucleotide polymorphism; T1DM: type 1 diabetes mellitus; T2DM; type 2 diabetes mellitus.

## Competing interests

The authors declare that they have no competing interests.

## Authors' contributions

PSR and CSVO performed the literature review and drafted the manuscript; FMAG supervised the project, helped in literature review and critically revised the manuscript for important intellectual content. AFR conceived and supervised the project, helped in literature review and critically revised the manuscript for important intellectual content. All authors have read and approved the final version of the manuscript.
